# The characteristics of overseas imported COVID-19 cases and the effectiveness of screening strategies in Beijing, China

**DOI:** 10.1186/s12879-021-06998-5

**Published:** 2022-01-17

**Authors:** Li Li, Cheng-Jie Ma, Yu-Fei Chang, Si-Yuan Yang, Yun-Xia Tang, Ling-Hang Wang

**Affiliations:** 1 Clinical and Research Center of Infectious Diseases, Beijing Ditan Hospital, Capital Medical University, Beijing, 100015 China; 2 Clinical Center for HIV/AIDS, Capital Medical University, Beijing, 100015 China

**Keywords:** Characteristics, Screening, COVID-19, Imported cases

## Abstract

**Background:**

In March 2020, the WHO declared the novel coronavirus outbreak a global pandemic. While great success in coronavirus disease 2019 (COVID-19) control has been achieved in China, imported cases have become a major challenge. This study aimed to describe the epidemiological and clinical characteristics of imported COVID-19 cases and to assess the effectiveness of screening strategies in Beijing, China.

**Methods:**

This retrospective study included all imported cases transferred to Beijing Ditan Hospital from 29 February to 20 March 2020 who were screened by both chest computed tomography (CT) and reverse-transcriptase-polymerase chain reaction (RT-PCR) at the initial presentation. Demographic, clinical and laboratory data, in addition to chest CT imaging, were collected and analysed.

**Results:**

In total, 2545 cases were included, among which 71 (2.8%) were finally diagnosed with laboratory-confirmed COVID-19. The majority 63 (88.7%) were from Europe. The most common initial symptoms were cough and fever, which accounted for 49.3% and 42.3%, respectively. Only four cases (5.6%) had lymphocytopenia, and thirteen cases (18.3%) demonstrated elevated levels of C-reactive protein (CRP). All cases had normal serum levels of procalcitonin (PCT). At initial presentation, among the 71 confirmed cases, 59 (83.1%) had a positive RT-PCR assay, and 35 (49.3%) had a positive chest CT. Twelve (16.9%) had a negative RT-PCR assay but a positive chest CT.

**Conclusions:**

A combination of RT-PCR and chest CT is an effective strategy for the screening of imported COVID-19 cases. Our findings provide important information and clinical evidence about the infection control of imported COVID-19 cases.

## Background

In December 2019, a cluster of patients with pneumonia of unknown cause occurred in Wuhan, Hubei Province, China [1–5]. The novel coronavirus, identified as the causative agent, is now formally named severe acute respiratory syndrome coronavirus 2 (SARS-CoV-2), and the disease caused by this novel coronavirus is called coronavirus disease 2019 (COVID-19) [6, 7]. Due to the lack of immunity against SARS-CoV-2 virus in humans, as well as its efficient transmission among humans, this virus spread rapidly across the world. Concerning COVID-19, the World Health Organization (WHO) raised the threat of the CoV epidemic to the "very high" level on February 28, 2020 [8].

Data provided by the WHO Health Emergency Dashboard (24 March 2020, 10:00 AM CET) report 332,930 confirmed cases of COVID-19 worldwide since the beginning of the epidemic [9]. Outside of China, the main endemic areas are Europe, the Americas and the Eastern Mediterranean region. Due to global economic integration, large numbers of Chinese people travel to endemic countries for trade, tourism, labour, study and other purposes. Subsequently, with the outbreak of COVID-19 abroad and the control of the epidemic in China, importation of COVID-19 from highly endemic areas into China is inevitable [10].

To address this new challenge, the rapid and accurate detection of imported cases is of great significance. In this study, we implemented border entry screening (BES) for overseas travellers and in-hospital screening for suspected cases. This provided us with a good opportunity to describe the characteristics of imported COVID-19 cases and to assess the effectiveness of the screening strategy in the first few months of the COVID-19 epidemic in Beijing, China.

## Methods

### Study design and subjects

A retrospective analysis was carried out on 71 overseas confirmed COVID-19 cases admitted to Beijing Ditan Hospital from 29 February to 20 March 2020, a designated hospital for the treatment of patients with COVID-19. All COVID-19 cases were diagnosed according to the Seventh Revised Trial Version of the Novel Coronavirus Pneumonia Diagnosis and Treatment Guidance [11]. A laboratory-confirmed COVID-19 case was defined as positive for SARS-CoV-2 nucleic acid on nasopharyngeal swabs and/or sputum specimens by reverse transcription polymerase chain reaction (RT-PCR) assays, which were performed by using TaqMan One-Step RT-PCR Kits from Da An Gene Co., Ltd. of Sun Yat-sen University (Da An; Guangzhou, China) and Shanghai BioGerm Medical Biotechnology Co., Ltd (BioGerm; Shanghai, China). The minimum detection limit of both kits was 500 copies/mL, and the specificity was 100% within the detection range.

### Screening process and data collection

**Fig. 1:**
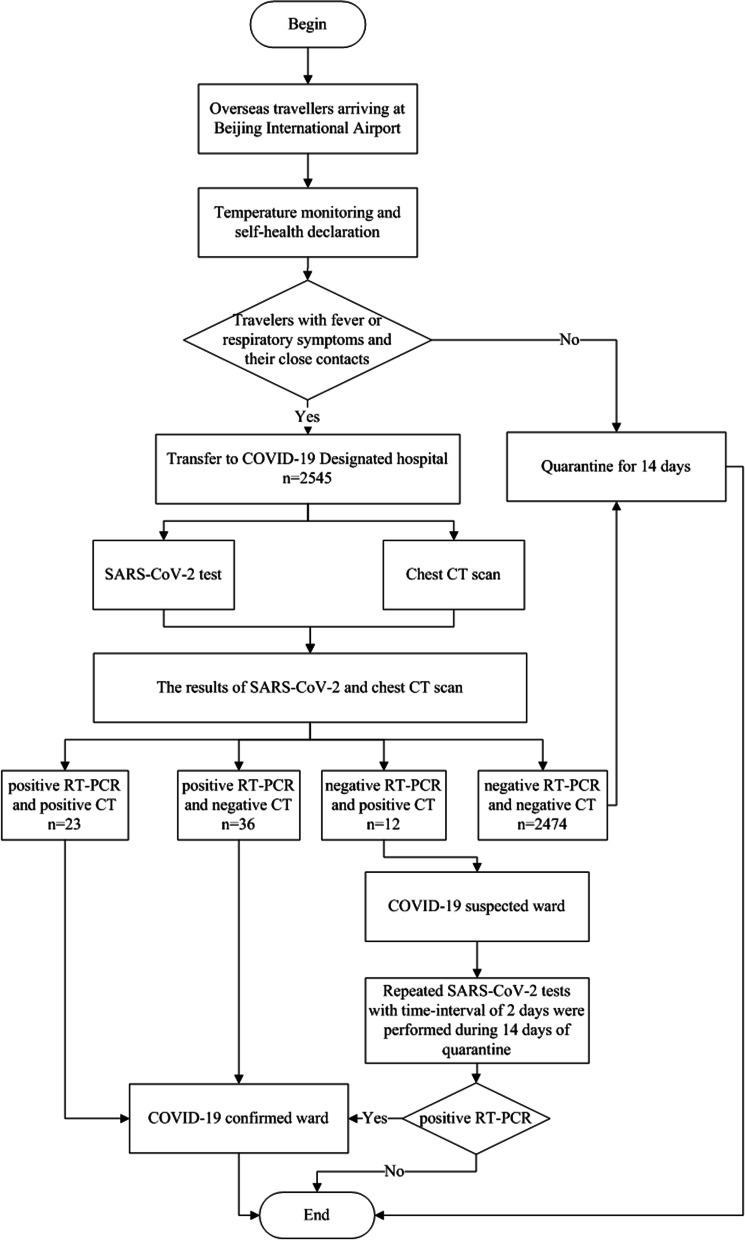
Flow chart of the screening process for overseas imported COVID-19 cases in the fever clinic. *CT* computed tomography; *SARS-CoV-2* severe acute respiratory syndrome coronavirus 2; *RT-PCR* reverse-transcriptase-polymerase chain reaction

Imported COVID-19 cases admitted to our hospital were detected using two detection routes in the fever clinic (Fig. 1):

1. Border entry screening: When an overseas flight arrived at Beijing International Airport, travellers were required to complete body temperature monitoring and self-health declarations during the customs check. Any traveller who was deemed to have symptoms of COVID-19 (including close contacts) was transferred to a COVID-19-designated hospital.
2. In-hospital screening: First, suspected travellers after initial screening at the airport would have been placed under respiratory isolation conditions. Then, in addition to a medical history and laboratory tests, SARS-CoV-2 tests and chest computed tomography (CT) were performed. The confirmed cases with positive RT-PCR results were admitted to the COVID-19 confirmed ward for further treatment. For highly suspected cases with a positive CT but a negative RT-PCR, they were quarantined in a single room in the COVID-19 suspected ward, and repeated RT-PCR tests were performed with a time interval of two days for further confirmation during 14 days of quarantine, especially for clustered cases. The diagnosis of COVID-19 was confirmed if they became positive for SARS-CoV-2. Otherwise, they were excluded as COVID-19.

Data were collected from each of the confirmed cases, including demographic data (i.e., sex, age, cluster, country of departure), clinical and laboratory results and chest CT features at the initial presentation. The laboratory results included a complete blood count, C-reactive protein (CRP) and procalcitonin (PCT). The measurement of PCT in the serum was performed by a VIDAS B.R.A.H.M.S. PCT assay (bioMérieux, Durham, NC), a one-step immunoassay sandwich method with enzyme-linked fluorescent assay detection that has a detection limit of 0.05 ng/ml.

### Statistical analysis

We described the categorical variables as frequencies and percentages (%) and continuous variables as mean and standard deviation (SD) for data with normal distribution (according to Kolmogorov–Smirnov test), otherwise as median and interquartile range (IQR) values. All statistical analyses were performed using SPSS (Statistical Package for the Social Sciences) version 22.0 software (SPSS Inc.).

## Results

### Demographic characteristics of the overseas imported COVID-19 cases

**Fig. 2:**
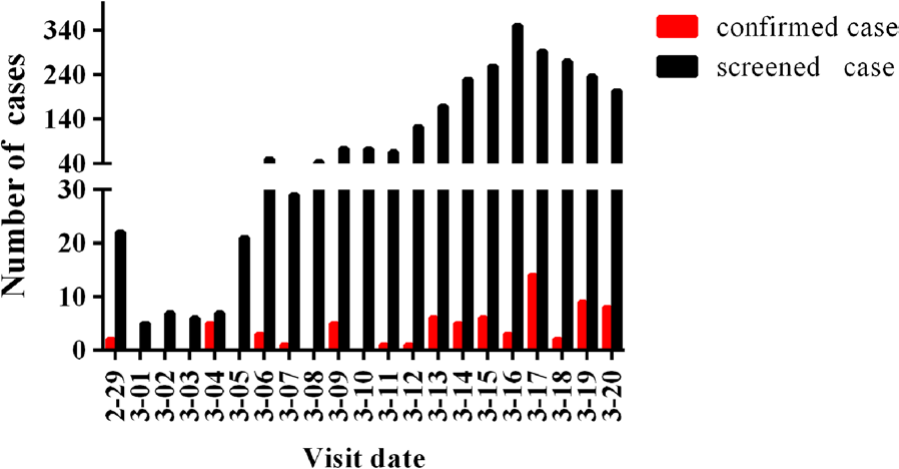
The daily screened and confirmed overseas imported cases in Beijing, China, from 29 February to 20 March 2020

From 29 February to 20 March 2020, 71 (2.8%) imported COVID-19 cases were identified from among 2545 overseas travellers screened at the emergency department of infectious diseases, Beijing Ditan Hospital, Capital Medical University. The demographic characteristics of the cases are described in Table 1. There were 27 (38.0%) males and 44 (62.0%) females. The median age was 24 years (IQR 20–39; range, 6–55 years). The imported cases were mainly from Europe. Among these, 22 cases (40.0%) were from Spain, followed by 17 cases (23.9%) from the United Kingdom and 16 cases (22.5%) from Italy. A total of 11 clusters occurred, accounting for 39.4% of all COVID-19 cases, including four from Italy, three from the United Kingdom, three from Spain, and one from Austria (Table 1). The period from 29 February to 10 March 2020 was characterized by low numbers of imported cases. From 11 March onward, there was a gradual increase in the number of imported cases, among which the maximum was 14 per day (Fig. 2).

**Table 1:** Demographic characteristics of the imported COVID-19 cases (N = 71)

Variables	Cases
Sex	
Male	27 (38.0%)
Female	44 (62.0%)
Age (years)	
Median (IQR)	24 (20–39)
≤ 19	12 (16.9%)
20–29	33 (46.5%)
30–39	10 (14.1%)
40–49	12 (16.9%)
≥ 50	4 (5.6%)
Clusters	11 (39.4%)
Country of departure	
Spain	22 (31.0%)
The United Kingdom	17 (23.9%)
Italy	16 (22.5%)
United States of America (USA)	5 (7.0%)
Hungary	3 (4.2%)
Austria	2 (2.8%)
Brazil	1 (1.4%)
Iran (Islamic Republic of)	2 (2.8%)
France	1 (1.4%)
Netherland	1 (1.41%)
Luxembourg	1 (1.41%)

Values are number (percentage) and median (IQR)

*IQR* interquartile range

### Clinical characteristics of the overseas imported COVID-19 cases

The median time from the illness onset to hospital admission was 4.0 days (IQR 2–7; range 11 h to 13 days). The most common symptoms at the onset of the illness were cough (35, 49.3%), followed by fever (30, 42.3%). Of 71 imported cases, 66 cases (93.0%) had more than one symptom. Among the other three symptomatic cases (4.2%), one only had fatigue and two only had low fever. Only two cases (2.8%) were asymptomatic but were members of family clusters (Table 2).

**Table 2:** Clinical characteristics of imported COVID-19 cases (N = 71)

Variables	Cases
Duration from onset to admission (d), median (IQR)	4 (2–7)*
Clinical on admission	
Cough	35 (49.3%)
Fever	30 (42.3%)
Sore throat	15 (21.1%)
Fatigue	14 (19.7%)
Headache	9 (12.7%)
Myalgia or arthralgia	7 (9.9%)
Shortness of breath	5 (7.0%)
Nausea or vomiting	2 (2.8%)
Diarrhea	1 (1.4%)
No sign or symptom	2 (2.8%)
Chest CT findings	
Bilateral pneumonia	19 (26.8%)
Unilateral pneumonia	16 (22.5%)
No abnormalities	36 (50.7%)

Values are number (percentage) and median (IQR)

*CT* computed tomography; *IQR* interquartile range

*Data of duration from onset to admission were available in 69 cases

On admission, the leucocytes were above the normal range in two cases (2.8%) and below the normal range in seven cases (9.9%). Four cases (5.6%) had lymphocytopenia. Platelets were above the normal range in eight cases (11.3%). C-reactive protein (CRP) was above the normal range in 13 cases (18.3%). All cases had normal serum levels of procalcitonin (Table 3).

**Table 3:** Laboratory findings of imported COVID-19 cases (N = 71)

Variables	Normal range	Cases			
		Median (IQR) or Mean (SD)	High No	Normal No	Low No
Blood routine					
Leucocyte count (× 10^9^/L)	4–10	5.67 (4.76–7.08)	2 (2.8%)	62 (87.3%)	7 (9.9%)
Lymphocyte count (× 10^9^/L)	1–5	1.69 ± 0.52	0 (0.0%)	67 (94%)	4 (5.6%)
Platelet count (× 10^9^/L)	100–300	236.00 ± 56.37	8 (11.3%)	63 (88.7%)	0 (0.0%)
Haemoglobin (g/L)	110–150 (female) 120–160 (male)	148.00 ± 15.76	16 (22.5%)	51 (71.8%)	4 (5.6%)
Infection-relation markers					
C-reactiveProtein (mg/L)	0–5	1.10 (0.40–3.40)	13 (18.3%)	58 (81.7%)	0 (0.0%)
Procalcitonin (ng/mL)	< 0.05	NA	0 (0.0%)	71 (100%)	NA

Values are number (percentage) and median (IQR) or mean (SD)

*IQR* interquartile range; *SD* standard deviation; *NA* not available

**Fig. 3:**
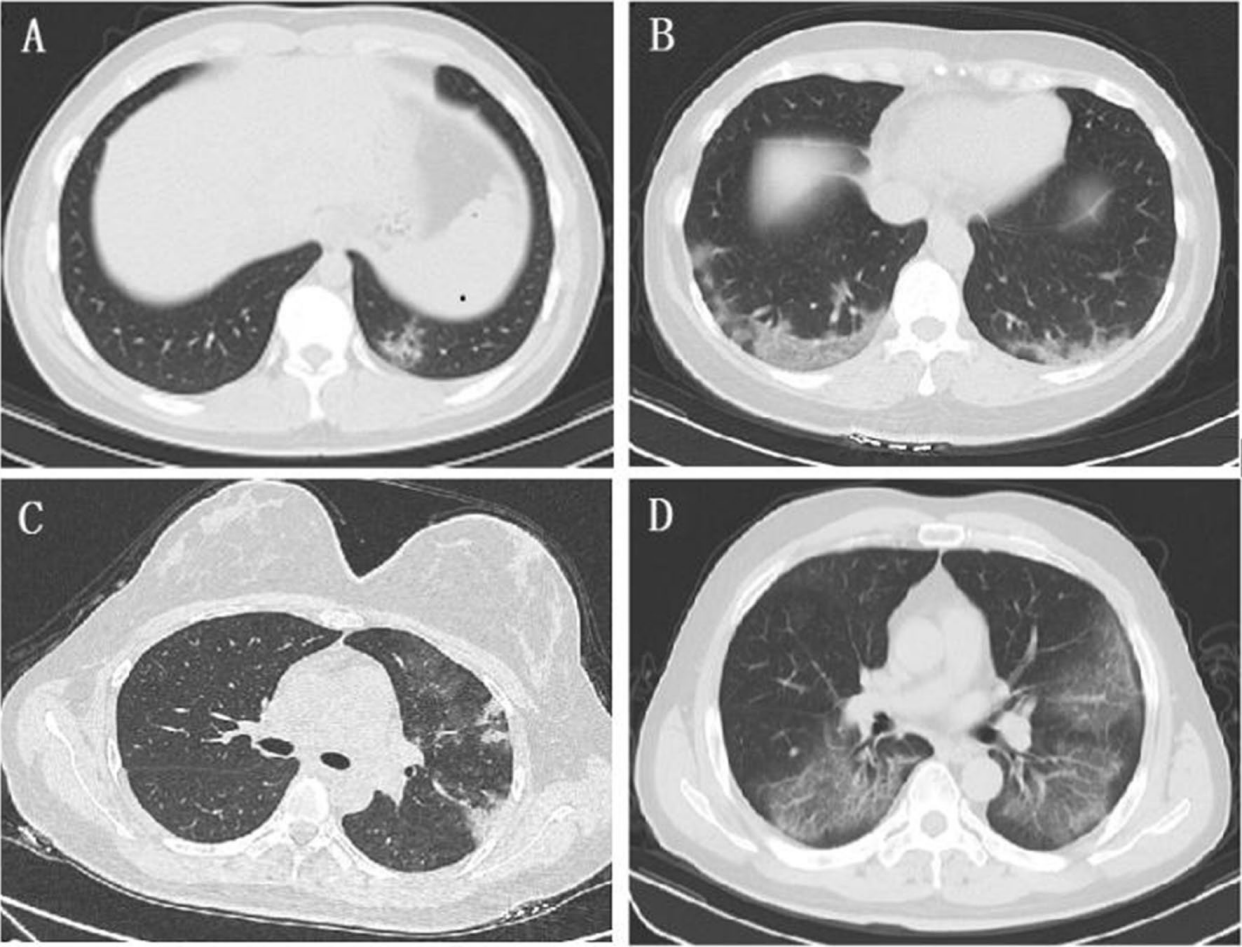
Chest CT images of COVID-19 cases. GGO, patchy ground-glass opacity; RR, respiratory rate; BP, blood pressure. **A** Chest CT image of a patient from The United Kingdom on the 1st day of hospitalization showing patchy ground-glass opacity (GGO) in the subpleural area of the left lower lobe (dry cough, no dyspnoea, no supplementary O_2_ required, SpO_2_ 98%, RR 18 times/min, BP 151/66 mmHg). **B** Chest CT image of a patient from America on the 1st day of hospitalization showing multiple patchy and spherical GGOs in the lower lobe of the bilateral lungs with interlobular septal thickening (fever, sore throat, no dyspnoea, no supplementary O_2_ required, SpO_2_ 96%, RR 20 times/min, BP 110/70 mmHg). **C** Chest CT image of a patient from The United Kingdom on the 1st day of hospitalization showing multiple patchy consolidations and GGOs in the left lung (fever, dry cough, no dyspnoea, no supplementary O_2_ required, SpO_2_ 97%, RR 18 times/min, BP 124/67 mmHg). **D** Chest CT image of a patient from Spain on the 1st day of hospitalization showing multiple patchy GGOs and early consolidation in the bilateral lungs (fever, dyspnoea, supplementary O_2_ required 2 L/min by nasal cannula, SpO_2_ 97%, RR 21 times/min, BP 127/86 mmHg)

According to the chest imaging findings at the initial presentation, of 71 imported cases, 35 cases (49.3%) showed abnormal chest CT images, consisting of 19 cases (26.8%) of bilateral pneumonia and 16 cases (22.5%) of unilateral pneumonia (Table 2), with typical findings of patchy ground-glass opacity (GGO) in the lungs (Fig. 3A, B). There were six cases (8.5%) of unilateral patchy consolidation and five cases (7.0%) of bilateral consolidation in the lungs (Fig. 3C, D). Thirty-six cases (50.7%) had no abnormalities in the parenchyma of either lung.

During the diagnostic procedure, the positive rates of the initial RT-PCR assay and chest CT imaging in our cohort were 83.1% (59/71) and 49.3% (35/71) for the diagnosis of COVID-19 cases, respectively. However, among the remaining twelve cases with initial nonpositive results, five of whom were eventually confirmed to have COVID-19 by two repeated RT-PCR tests, four were confirmed by three tests, one was confirmed by four tests, and two were confirmed by five tests.

### The effectiveness of the screening strategy

From 29 February to 20 March 2020, with the combination of RT-PCR and CT, 2.8% (71/2545) of entry screening cases were detected and quarantined in time. However, those who were excluded as COVID-19 were repeatedly tested for SARS-CoV-2 during 14 days of quarantine and all SARS-CoV-2 results were negative as confirmed by telephone follow-up with doctors at designated places. At the same time, the local Centers for Disease Control and Prevention (CDC) had not reported any new confirmed cases in this population.

## Discussion

The COVID-19 epidemic was dominated by overseas imported cases between March and June 2020 [12], which became a new challenge for the control of COVID-19 in Beijing, China. In this retrospective study, 2.8% (71/2545) of entry screening cases were diagnosed with COVID-19 by a combination of RT-PCR and chest CT. The RT-PCR assay of the respiratory specimens diagnosed most COVID-19 cases (83.1%). A positive chest CT was found in 49.3% of cases; however, among these, 34.3% were also diagnosed by repeated RT-PCR.

Focusing on the period from 29 February to 20 March 2020, there was a consistent increase in the number of imported COVID-19 cases from overseas into Beijing, China. The countries of origin of the imported cases reflected the patterns of SARS-CoV-2 activity in that country at that time. Screening should focus on travellers coming from countries with high COVID-19 activity. The first imported case seen in Beijing was from Iran on 29 February 2020, corresponding to the severity of the COVID-19 outbreak that occurred in Iran at the same time. However, over the course of the next few days, the case exporting regions expanded, and cases from around the world were identified. This demonstrated the global spread of the disease during the development of the pandemic. This also suggests that a detailed epidemiological history is of paramount importance for the early detection of COVID-19 patients.

All imported cases were screened first at customs via temperature monitoring and self-health declarations and then transferred to the emergency department of infectious diseases in Beijing Ditan Hospital. In this study, the most common symptoms were cough and fever, similar to the cohorts reported in the currently available literature [13–15]. However, compared with those with SARS-CoV (99%) and MERS-CoV (98%) [16], fever was less frequent in those with SARS-CoV-2. Notably, two cases in our study were asymptomatic but were members of family clusters, but they were positive for SARS-CoV-2 nucleic acids, indicating that contacts of the confirmed cases within their clusters cannot be ignored during screening [17]. It is vital to strengthen the surveillance and tracing of this population.

Currently, RT-PCR tests are the gold standard diagnostic tool for COVID-19 [18, 19]. In this study, all patients received the SARS-CoV-2 test at initial presentation. The positive rate of the initial RT-PCR assay for respiratory samples was 83.1%, which is consistent with a previous report [20]. In fact, from the results of this study, 16.9% (12/71) of the patients with negative RT-PCR results but a positive chest CT were diagnosed with COVID-19 through repeated RT-PCR. These negatives could result from improper sampling techniques or a low viral load in the area sampled [21, 22]. Therefore, for patients with a high clinical suspicion, specimens should be continuously collected for multiple tests to avoid a missed diagnosis.

Previous studies have shown that chest CT scans are of great significance to screen suspected cases of COVID-19 [23]. In the early stage, there was ground-glass opacification with or without consolidative abnormalities, especially with a peripheral distribution. In severe cases, lung consolidation may occur, but pleural effusion is rare [24]. In our study, nearly half of the imported cases showed typical CT features consistent with the study by Huang et al. [25]. For these cases, chest CT may be used for clinical staging of the diseases. In addition, 16.9% (12/71) had initial positive chest CT scans prior to the initial negative RT-PCR results, indicating that chest CT, where it is available, may identify cases that have progressed to pneumonia but are no longer shedding virus from the upper respiratory tract. Notably, normal chest CT imaging was found in 36 (50.7%) cases compared to 17% in a recent study by Pan et al. [26]. The imaging features of COVID-19 are diverse and depend on the stage of infection after the onset of symptoms. A retrospective analysis of chest CT in 121 patients with COVID-19 by Bernheim et al. [27] showed more frequent normal CT findings (56%) in the early stages of the disease (0–2 days). Therefore, a normal result from the initial CT scan does not completely rule out COVID-19.

There are several limitations to our study. First, due to the limited number of patients, our conclusions need to be further verified in large samples and multicentre data. Second, due to time constraints, those who were excluded as having COVID-19 at the initial presentation were not followed up for longer periods of time. Therefore, continued attention needs to be paid to the reports of the local CDC about COVID-19 outbreaks for further verification.

## Conclusions

Currently, SARS-CoV-2 continues to spread globally. To accurately detect imported COVID-19 cases, the following aspects should be focused: (1) Strengthen the surveillance of overseas COVID-19 outbreaks. Airport customs personnel and doctors in hospitals should update the epidemic situation abroad synchronously. (2) Strengthen the understanding of the clinical characteristics of the imported cases and use combined screening.

## Data Availability

Data of the study can be available upon request from Ling-hang Wang.

## Abbreviations

COVID-19: Coronavirus disease 2019
SARS-CoV-2: Severe acute respiratory syndrome coronavirus 2
CT: Computed tomography
RT-PCR: Reverse-transcriptase-polymerase chain reaction
CRP: C-reactive protein
PCT: Procalcitonin
WHO: World Health Organization
BES: Border entry screening
SD: Standard deviation
IQR: Interquartile range
GGO: Ground-glass opacity
CDC: Centers for Disease Control and Prevention
